# Non-Surgical Treatment of Symptomatic, Oblique Strabismus: A Simplified Approach

**DOI:** 10.22599/bioj.318

**Published:** 2023-12-08

**Authors:** Alex Christoff

**Affiliations:** 1 The Wilmer Eye Institute at Johns Hopkins Hospital, 600 North Wolfe Street Wilmer 233, Baltimore, Maryland 21287, US

**Keywords:** Prism, Press-On Prism, Prism and Cover Test, Diplopia

## Abstract

**Purpose:** Determining the correct power and orientation of prism to be prescribed for patients with symptomatic, oblique-angle strabismus can be challenging and confusing, prone more to clinician gestalt than science or methodology. The author shares a simplified, approach not previously described in the scientific literature that utilizes commercially available equipment and freely available on-line prism calculators for choosing the correct Press-On™ prism power, positioning the prism correctly on the spectacle lens, and ultimately determining the correct prism prescription to be incorporated into the patient’s spectacles.

## Introduction

Strabismus surgery is an effective treatment in adults with symptomatic horizontal and vertical strabismus. However, there are times when surgery is not a suitable option, such as when there is a concern for anesthetic risk, when the deviation is dynamic or unstable—as seen in patients recovering from ischemic neuropathies, from restrictive strabismus associated with Grave’s orbitopathy, and in neuro-muscular disorders, such as myasthenia gravis—or when the patient declines.

**Figure 1:**
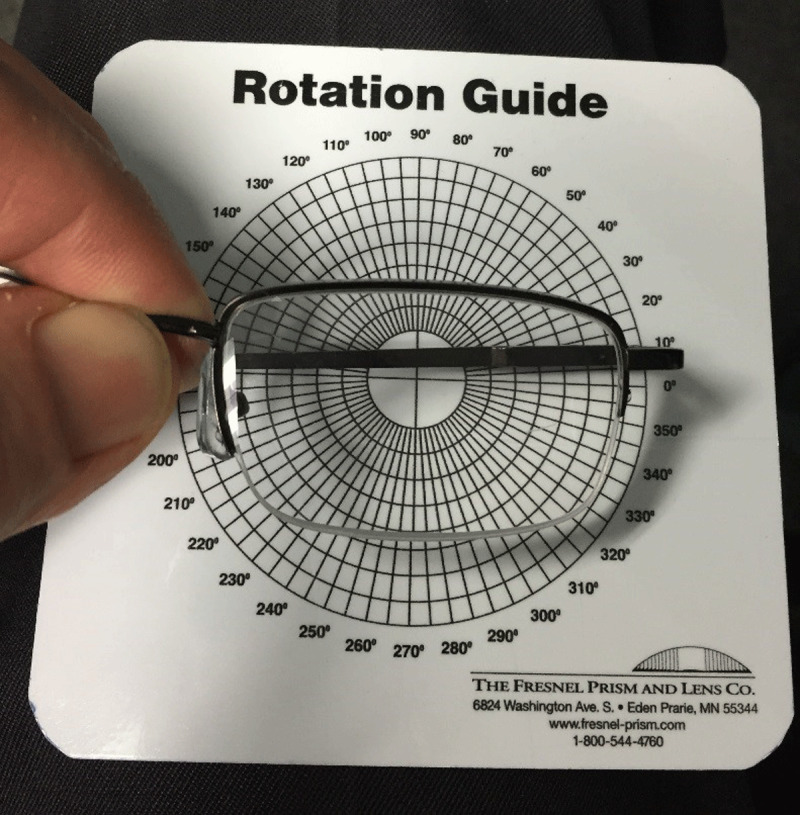
A 360-degree rotation guide used to orient the appropriate power prism on the spectacle lens (The Fresnel Prism and Lens Company, Eden Prairie, MN). The rotation guide is positioned at the geometric center of the spectacle lens, not the optical center.

Techniques to measure and treat combined horizontal and vertical strabismus with a temporary prism have been described by others (Anilkumar and Narendran 2022; Kortvelesy 2008). These earlier methods involve quantifying the vertical and horizontal components of the deviation using prism and alternate cover testing (PACT), and then applying the Pythagorean theorem to calculate a diagonal, oblique prism equivalent. This is a time-consuming step and requires the clinician to calculate the hypotenuse of a simple right triangle, whose legs are defined by the magnitudes of the horizontal and vertical strabismus measured in the clinic. One can also consult reference tables published over 50 years ago that provide a resultant power and angle for an oblique prism in a limited range of mixed horizontal and vertical strabismus cases (Reinecke 1997). A 360-degree prism rotation guide (The Fresnel Prism and Lens Company, Eden Prairie, MN) is then used to orient the preferred temporary prism at the correct axis on the spectacle lens (Figure 1). This step is key to successful resolution of oblique diplopia with a temporary prism, but traditionally many orthoptists have done this by hand, using trial and error.

This paper presents an alternative and more modern technique, which facilitates an efficient and more accurate determination of the correct power and rotation axis of a temporary prism for mixed horizontal and vertical strabismus by using an online prism calculator.

## Methods

The author’s described method allows for fast and efficient calculation of complex prism prescriptions to be incorporated into prism spectacles. All is accomplished in under a minute with the patient’s exam data.

**Figure 2:**
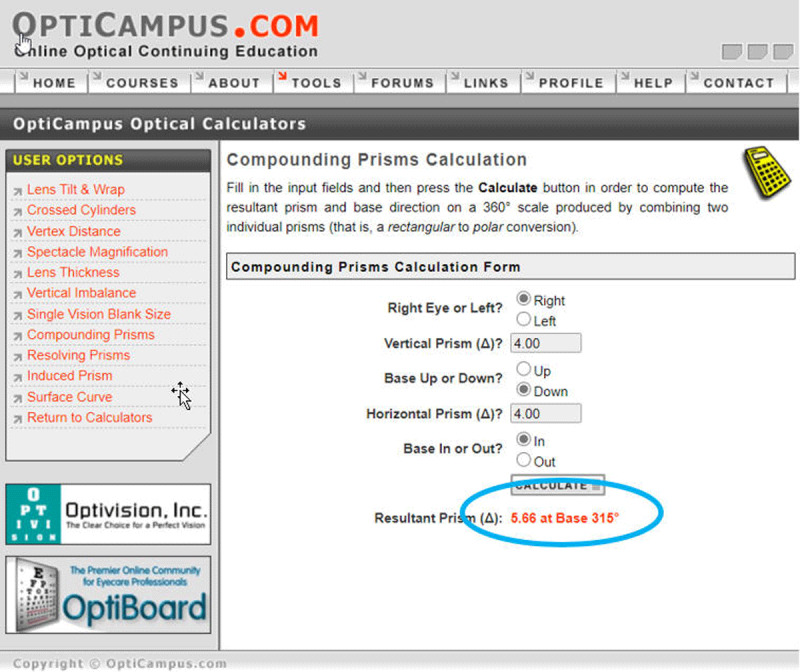
Opticampus.com compounding prism calculator used to calculate prism power and axis notation of an obliquely placed temporary prism. The clinician selects the appropriate eye and enters prism power and base direction for the vertical and horizontal strabismus measured by cover testing to obtain the final prism rotation axis based on the 360-degree system.

**Figure 3:**
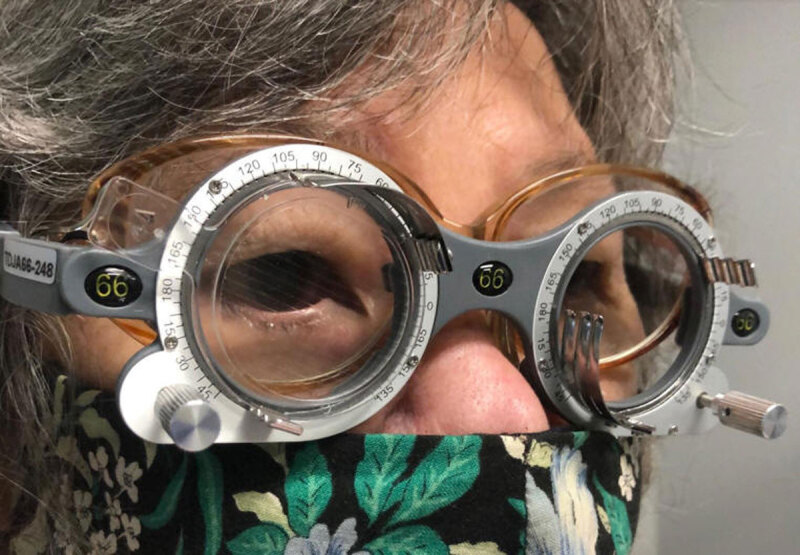
Obliquely placed plastic Fresnel ring prism (The Fresnel Prism and Lens Company, Eden Prairie, MN), shown base up and in at 80 degrees on the right in 66 mm trial frames over the patient’s habitual spectacles.

**Figure 4:**
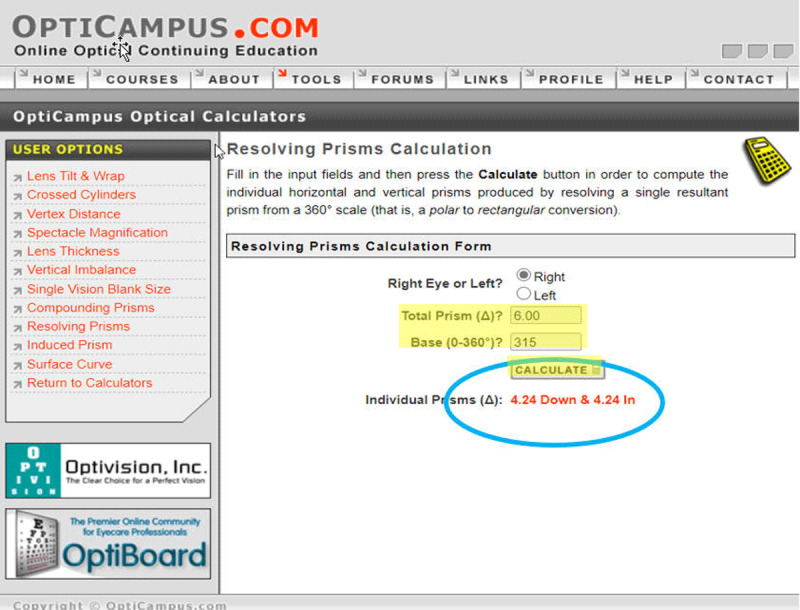
Opticampus.com resolving prism calculator used to calculate individual horizontal and vertical spectacle prisms from an obliquely placed temporary prism.

The process begins by quantifying the horizontal and vertical deviations in the primary gaze position with prism and cover testing (PACT). For example, a patient measures exotropia (XT) 15 prism diopters (PD) and right hypertropia (RHT) 10 PD in the primary gaze position at distance fixation. The examiner then uses these values, 15 PD base in and 10 PD base down over the right eye (OD) to fill in the required fields of the *compounding prism calculator* (Figure 2; OptiCampus 2023). A calculated prism power and orientation of 18 PD at 326 degrees (base in and down OD) is obtained. Because trial prism powers are not available in 18 PD increments, the clinician will have to determine if 15 PD or 20 PD is most appropriate. The author recommends using prism trial rings (The Fresnel Prism and Lens Company, Eden Prairie, MN) in a trial frame for this and asking the patient to fine-tune the prism rotation axis with the cylinder adjustment knob until the diplopia resolves. The rings are round, thin, flat plastic prisms available in two 7-ring boxed sets. The first set includes powers of 2, 3, 4, 5, 6, 8, and 10 PD. The second set contains 12, 15, 20, 25, 30, 35, and 40 PD. These round prism lenses clip into a standard 38 mm trial frame, or the orthoptist can purchase a boxed set of five lightweight trial frames commercially (Good-Lite Company, Elgin, IL). Adult-sized trial frames are available in pupillary distances of 62, 64, 66, 68, and 70 mm and are designed to hold the standard 38 mm trial lens; three lenses anterior to the frame and a fourth lens behind the frame. They fit over every contemporary adult spectacle frame the author has ever encountered in the clinic, so the clinician does not have to transfer the patient’s spectacle prescription to the trial frame and then add the prism. Each frame also has adjustment knobs to rotate the prism axis of the anterior lens, and temple length is adjustable. For patients already wearing glasses, insert the flat plastic Fresnel prism lens into trial frames of an appropriately sized trial frame and fit these over the patient’s spectacles (Figure 3). The patient fine-tunes the prism rotation axis with the trial frame cylinder adjustment knob, repositioning the base of the prism slightly until the diplopia or blur has resolved to their satisfaction. For new spectacle prescriptions placed in the trial frames, the examiner will have to maintain the correct cylinder axis of any astigmatic correction used as the patient rotates the position of the prism with the cylinder axis adjustment knob. This challenge can be addressed by positioning the astigmatic lens in the posterior, non-rotating position behind the trial frame, closest to the patient’s face, leaving the spherical lens component of the prescription free to rotate with the plastic prism lens being adjusted by the patient. Having arrived at an appropriate prism power and base orientation, the clinician should make note of the final axis position, again, based on the 360-degree scale for that eye (explained in detail later in this article) or mark the base position on the patient’s spectacle lens with a wax pencil or non-permanent marker. This will enable an accurate placement of the temporary prism to be applied to the posterior surface of the spectacle lens. Once the temporary Press-On® prism has been worn successfully without diplopia outside the clinic, consideration can be given to a more permanent, ground-in spectacle solution. For this task, the author uses the *resolving prism calculator* (OptiCampus 2023); entering the temporary prism information into the required data fields (Figure 4), the author can calculate the final vertical and horizontal prism power and prism base orientation for one lens. In the previous example, if diplopia resolved with 15 PD in and down at 330 degrees on the right, filling in the appropriate fields in the resolving prism calculator results in a recommended spectacle prism prescription of 7.50 PD down and 13.00 PD in. This can be incorporated into just the right lens, or preferably divided appropriately between both lenses.

## Discussion

**Figure 5:**
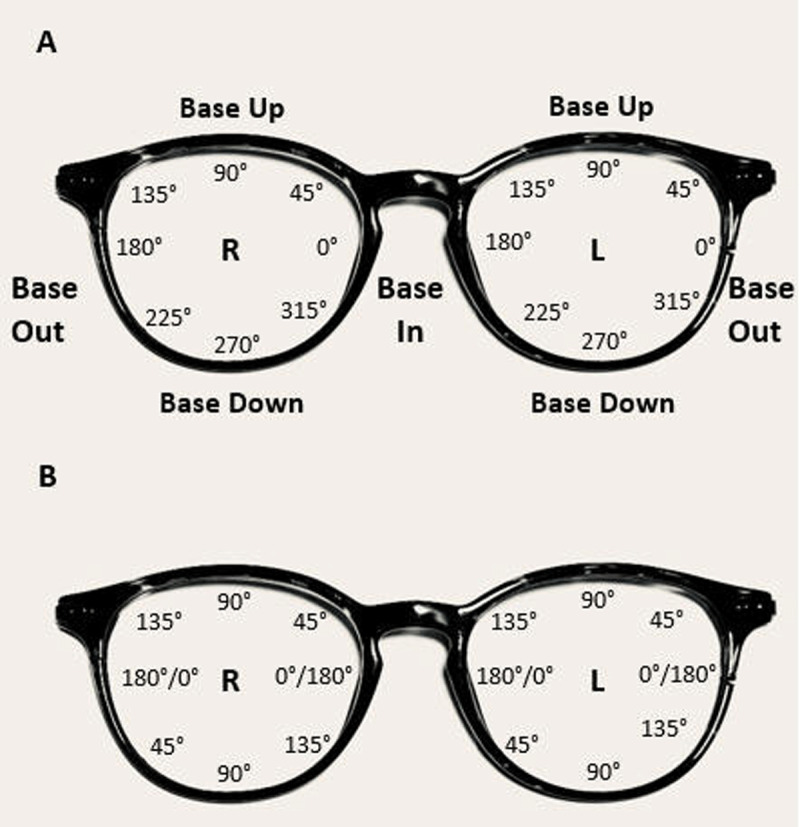
A: Illustration of 360-degree prism base notation in spectacles compared with B: 180-degree cylinder axis notation in spectacles.

**Figure 6:**
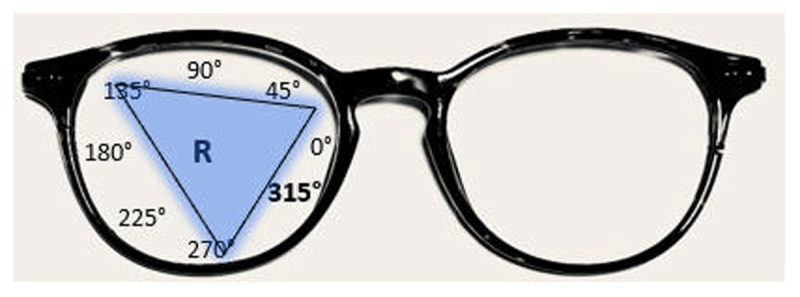
Graphic showing base orientation of a prism oriented base in and down on the right lens, in this example, the base is positioned at 315 degrees, not 45 degrees.

The prism calculators referenced in this article are available at OptiCampus.com. This is an online, continuing education resource that offers free access to several optical dispensing tools. Web hosting for OptiCampus.com is provided by OptiBoard, and web database programming is maintained by Optivision, Inc. The conversion from an obliquely placed temporary prism to the horizontal and vertical prism is accomplished quickly and easily on the website. Alternatively, it may be used the other way around—from known horizontal and vertical spectacle prism strength, the *compounding prism* calculator (Figure 2) can arrive at an equivalent temporary oblique prism power and rotation axis. It is important to point out that trial frames and phoropters use a repeating 180-degree system for cylinder axis notation (Figure 5b). However, prism base notation is not confined to the same two principal meridians located 90 degrees apart as is the case with spherocylindrical lenses (Riordan-Eva, 2002–2003). Calculations for determining oblique prism orientation incorporated into spectacle lenses mathematically must be based on a 360-degree compass rose system. From the clinician’s perspective facing the patient, the 360-degree compass rose starts from the 3 o’clock position in front of each eye and moves counterclockwise around the eye. This orientation is different for the right eye and the left eye (Figure 5a). Facing the patient, 180 degrees is a temporal position for the right lens and a nasal position for the left lens. But in either eye, the 3 o’clock position corresponds to 0/360 degrees, 12 o’clock corresponds to 90 degrees (base up), 9 o’clock corresponds to 180 degrees, and 6 o’clock corresponds to 270 degrees (base down). Applied to spectacles, the correct prism base notation of an example prism is shown in Figure 6, base in and down at 315 degrees, not 45 degrees, on the right. Documented inaccurately, subsequent calculations to determine permanent, spectacle prism will be incorrect.

Resolving diplopia associated with vertical strabismus assumes no significant cyclotorsion, which may preclude fusion with prisms in free space. Flodin and colleagues (2020) reported that subjective intorsion greater than only 7 degrees or subjective extorsion greater than 9 degrees could preclude fusion with prism offset in their series of 120 healthy adult volunteers.

For small, manifest angles of strabismus, the clinician should pay particular attention to the simultaneous prism and cover test (SPCT) results. This measured angle of manifest strabismus can be a useful starting point from which to choose the prism power required to resolve a patient’s diplopia. In an unpublished series of 248 consecutive orthoptic clinic patients seen by the author in a 3-year period, a study approved by the Johns Hopkins Institutional Review Board and accepted as a poster at the 46th Annual Meeting of the American Association of Pediatric Ophthalmology and Strabismus (AAPOS), 36 patients with diplopia had both SPCT and PACT measurements performed. Mean SPCT measurement was 7 PD (median 5 PD, range 1–20 PD, 95% CI), and mean PACT was 13 PD (median 11 PD, range 2–30 PD, 95% CI). Mean prism power dispensed was 8 PD (median 6 PD, range 1–25 PD, 95% CI). The difference of mean SPCT versus mean dispensed was –1.31 PD (95% CI: –2.81 PD, 0.19 PD; *p* = 0.086) suggesting a close relationship between the SPCT measurements and Press-On prism power applied. There was a large difference between mean PACT versus mean dispensed of 5 PD (95% CI: 3.16 PD, 6.16 PD; *p* < 0.001). Twenty-four patients (66%) remained in prism one year later. Eight patients (23%) elected to have strabismus surgery, six with horizontal strabismus (4 ET/1 XT), and two with vertical strabismus. For the surgery group, mean SPCT was 11 PD (median 12 PD, range 1–20 PD), mean PACT was 19 PD (median 20 PD, range 2–25 PD), and mean prism power dispensed for the surgery group was 13 PD (median 12 PD, range 5–25 PD). This data suggests that for any patient for whom prism is being considered to treat symptomatic strabismus, prism powers in any orientation of much more than 12 PD are not well tolerated long term, and strabismus surgery is probably a better option. The lower starting values of required prism power in the case series above can be explained by residual fusional convergence, divergence, or vertical vergence amplitudes, and accordingly, the required prism power is often far less than the maximum deviation measured after full dissociation with PACT. Thus, for patients demonstrating fusion with symptomatic phorias or intermittent strabismus, start with lower prism values and increase as necessary to resolve symptoms.

## Conclusion

Commercially available supplies and freely available online calculators are now available to assist in non-surgical treatment of symptomatic combined horizontal and vertical strabismus quickly and efficiently, allowing the clinician to choose the appropriate prism power, position the prism correctly on the spectacle lens, and accurately determine the correct prism prescription to be incorporated into the patient’s spectacles.
